# Molybdenum-Induced Regulation of Antioxidant Defense-Mitigated Cadmium Stress in Aromatic Rice and Improved Crop Growth, Yield, and Quality Traits

**DOI:** 10.3390/antiox10060838

**Published:** 2021-05-24

**Authors:** Muhammad Imran, Saddam Hussain, Longxin He, Muhammad Furqan Ashraf, Muhammad Ihtisham, Ejaz Ahmad Warraich, Xiangru Tang

**Affiliations:** 1 State Key Laboratory for Conservation and Utilization of Subtropical Agro-Bioresources, College of Agriculture, South China Agricultural University, Guangzhou 510642, China; muhammadimran@scau.edu.cn (M.I.); helx@stu.scau.edu.cn (L.H.); 2 Scientific Observing and Experimental Station of Crop Cultivation in South China, Ministry of Agriculture and Rural Affairs, Guangzhou 510642, China; 3 Guangzhou Key Laboratory for Science and Technology of Aromatic Rice, Guangzhou 510642, China; 4 Department of Agronomy, University of Agriculture Faisalabad, Punjab 38040, Pakistan; shussain@uaf.edu.pk (S.H.); ewarraich@uaf.edu.pk (E.A.W.); 5 College of Life Sciences, South China Agricultural University, Guangzhou 510642, China; furqan2210uaf@scau.edu.cn; 6 College of Landscape Architecture, Chengdu Campus, Sichuan Agricultural University, Wenjiang 611100, China; ihtisham@sicau.edu.cn

**Keywords:** molybdenum, cadmium stress, photosynthetic pigments, oxidative damage, quality characters, fragrant rice

## Abstract

Cadmium (Cd) stress causes serious disruptions in plant metabolism, physio-biochemical responses, crop yield, and grain quality characteristics. A pot experiment was conducted to investigate the role of molybdenum (Mo) in mitigating Cd-induced adversities on plant growth, yield attributes, and grain quality characteristics of a popular aromatic rice cultivar ‘Xiangyaxiangzhan’. The Mo was applied at 0.15 mg kg^−1^ soil in both control (no Cd) and Cd-contaminated (100 mg kg^−1^) soils. A treatment with Mo-free (−Mo) soil was also maintained for comparison. The results showed that Cd toxicity significantly (*p* < 0.05) reduced plant dry biomass, grain yield, photosynthetic efficiency, and pigment contents, and impaired chloroplast ultra-structural configuration and simultaneously destabilized the plant metabolism owing to higher accumulation of hydrogen peroxide, electrolyte leakage, and malondialdehyde contents. However, Mo supply improved grain yield and 2-acetyl-1-pyrroline content by 64.75% and 77.09%, respectively, under Cd stress, suggesting that Mo supply mitigated Cd-provoked negative effects on yield attributes and grain quality of aromatic rice. Moreover, Mo supply enhanced photosynthesis, proline, and soluble protein content, and also strengthened plant metabolism and antioxidant defense through maintaining higher activities and transcript abundance of ROS-detoxifying enzymes at the vegetative, reproductive, and maturity stages of aromatic rice plants under Cd toxicity. Collectively, our findings indicated that Mo supply strengthened plant metabolism at prominent growth stages through an improved enzymatic and non-enzymatic antioxidant defense system, thereby increasing grain yield and quality characteristics of aromatic rice under Cd toxicity.

## 1. Introduction

Aromatic rice, the finest quality rice, is recognized around the world for its unique fragrance and taste [1]. The rice milling standards could be measured by head rice rate, milling recovery percentage, and milled rice rate, which are directly connected to market values, whereas the rice chalkiness percentage and grain protein contents belong to cooking characteristics of aromatic rice [2].

Cadmium (Cd) toxicity is one of the third major contaminants in agricultural soils across the globe and is considered to be the only metal that poses health hazards to humans and animals at a plant tissue concentration that is usually non-phytotoxic [3,4]. In paddy soils, Cd is taken up and moved to above ground components by plant roots and affects natural plant metabolism, morpho-physiological characteristics, crop growth, and productivity [5]. Specifically, Cd toxicity leads to stunted plant growth, photosynthesis disturbance, reductions in chlorophyll biosynthesis, deformation of cellular structures, and higher production of reactive oxygen species (ROS), disturbance in cellular functions, and ultimately plant death [6,7,8].

Molybdenum (Mo), an indispensable microelement, is involved in multiple metabolic and cellular processes in higher plants [9,10]. It plays a key role in different plant physio-biochemical processes, such as photosynthesis, chloroplast configuration and ultra-structural integrity, and chlorophyll biosynthesis and also acts as a stress-resilient element to enhance the oxidative stress tolerance under salinity [11], drought [12], low temperature [13], and ammonium toxicity [14]. Several recent studies have documented the beneficial functions of Mo in alleviating Cd toxicity in *Brassica napus* L. [15] and *Cannabis sativa* L. [16]. The Mo-induced mitigation of Cd toxicities has been ascribed to following four mechanisms: (i) obstructing the absorption and translocation of Cd and manipulating its fractions in plant cells; (ii) repairing damaged cell membranes, ultra-structures of chloroplasts, and reinforcing the photosynthetic system; (iii) regulating the uptake of essential elements; and (iv) activating the antioxidant defense systems (both enzymatic and non-enzymatic) in order to detoxify ROS [4].

However, little is known regarding the effects of Mo on plant physio-biochemical processes, grain yield, and grain quality traits of aromatic rice plants under Cd toxicity. Therefore, the present study was conducted to investigate the protective effects of Mo supply against Cd toxicity on plant growth, physio-biochemical processes, and antioxidant defense responses of aromatic rice plants at three prominent growth stages (vegetative, reproductive, and maturity stages), and consequently examine the Mo-induced enhancement in rice yield and grain quality characteristics under Cd toxicity.

## 2. Materials and Methods

### 2.1. Experimental Location, Treatments, and Crop Husbandry

A pot experiment was performed during July–November 2020 at the Experimental Research Farm (113°21′ E, 23°14′ N), College of Agriculture, South China Agricultural University (SCAU), Guangzhou, China, in a rain-sheltered wire house under open-air conditions. The soil used in this experiment was under paddy cultivation for many years. The above-soil layer (0–20 cm) was used to fill the pots (10 kg air-dried soil in each pot with 25 cm in height and 32 cm in diameter). The essential chemical properties of the soil used in this experiment are shown in Table S1. The experimental treatments consisted of Mo (0 and 0.15 mg kg^−1^ soil) in the form of ammonium molybdate [(NH_4_)_6_Mo_7_O_24_·4H_2_O]) and Cd (0 and 100 mg kg^−1^ soil) supplied as CdCl_2_·2.5 H_2_O. About two weeks before transplantation, the treatment concentrations were carefully mixed in the soil of respective pots and a water layer (2–3 cm) was maintained over the soil surface to create puddled-like and anaerobic conditions.

The seeds of aromatic rice cultivar “Xiangyaxiangzhan” were collected from South China Agricultural University, Guangdong, China. This aromatic rice cultivar is locally renowned for its special aroma-producing quality and therefore widely cultivated by local farmers of South China on a commercial scale. However, previous studies have reported that heavy metals (e.g., Cd, Pb) significantly affect the grain yield, aroma, and other quality traits of this aromatic rice cultivar [2,17]. The uniform and well-grown rice seedlings (22-days-old) were transplanted in soil-filled pots (3–4 seedlings per hill and 5 hills per pot) by following a cross mark (+) type of planting pattern (four seedlings at a side each and the fifth in the middle of the pot). All the experimental pots were supplied with 2.25, 3.33, and 1.35 g N-P-K in the form of urea, phosphorus pentoxide, and potassium oxide, respectively.

### 2.2. Sampling

At three prominent growth stages (vegetative, reproductive, and maturity stages), fresh plant leaves were sampled and well-preserved (−80 °C) for the assessment of photosynthetic pigments, various physio-biochemical analysis, antioxidant enzyme activities, and qRT-PCR quantification. At maturity, plants were harvested to measure dry biomass, grain yield, and 2AP content to analyze the qualitative characteristics of aromatic rice grains.

### 2.3. Estimation of Photosynthetic Efficiency, Pigment Contents, and Transmission Electron Microscopy

For the photosynthetic measurement, one fully expanded mature leaf from the top was used per plant and five plants were selected in each replication of a particular treatment and the results were averaged. The photosynthetic rate was recorded with a portable photosynthetic system (LI- 6400xt, Li-Cor, Inc., Lincoln, NE, USA). The photosynthetic pigments were determined in accordance with the method described in previous studies [18,19]. Fresh leaf samples were randomly collected from different aromatic rice plants for TEM analysis. Small sections of leaves 1–3 mm in length were fixed in 4% glutaraldehyde (*v/v*) in 0.2 mol/L PBS (sodium phosphate buffer, pH 7.2) for 6–8 h and post-fixed in 1% OsO_4_ (Osmium (VIII) oxide) for 1 h, then in 0.2 mol/L PBS (pH 7.2) for 1–2 h. Dehydration was performed in a graded ethanol series (50%, 60%, 70%, 80%, 90%, 95%, and 100%) followed by acetone, then samples were filtrated and embedded in Spurr’s resin. Ultra-thin sections (80 nm) were prepared and mounted on copper grids for viewing under a transmission electron microscope (TALOS L120C) [20].

### 2.4. Measurement of Soluble Protein and Proline Contents

The protein contents in leaves were estimated according to the method of [21] using G-250. The fresh leaf samples (0.2 g) were homogenized in 50 mM sodium phosphate buffer (pH 7.0) containing 2% polyvinylpyrrolidine-40 (PVP-40) and 1 mM EDTA-Na2. The reaction mixture was centrifuged at 10,000× *g* for 15 min at 4 °C. The absorbance of the reaction mixture was read at 595 nm in triplicate and final protein contents were expressed as mg g^−1^ FW. The proline contents were estimated according to the method of [22] using ninhydrin. The reaction mixture was extracted with 5 mL toluene and the absorbance of the red chromophore in the toluene fraction was measured at 520 nm. The amount of proline was estimated by comparing with a standard curve and expressed as µg g^−1^ FW.

### 2.5. Measurement of Reactive Oxygen Species and Lipid Peroxidation

Fresh leaf samples (0.2 g) at different growth stages were crushed in liquid nitrogen and homogenized with 1 mL of 0.1% trichloroacetic acid (TCA) and centrifuged at 12,000× *g* for 20 min (4 °C) for the measurement of H_2_O_2_ content in aromatic rice plants. The reaction mixture consisted of 0.5 mL of potassium phosphate buffer (pH 6.8, 100 mM), 1 mL potassium iodide (1M), and 0.5 mL supernatant. The H_2_O_2_ contents were measured by spectrophotometer (UV-VIS 2550, Shimadzu, Japan) at 390 nm [23]. The malondialdehyde (MDA) contents in leaves at different growth stages of aromatic rice were estimated by a prior method [24]. Fresh leaf samples (0.2 g) were homogenized in 2 mL 0.5% thiobarbituric acid (TBA) solution in 10% trichloroacetic acid (TCA) and boiled in the water bath at 100 °C for 30 min. The boiled samples were then cooled down in an ice bath and centrifuged at 4000× *g* for 15 min. The absorbance of the reaction mixtures was read at 532, 600, and 450 nm in triplicate. The MDA content in the reaction solution was calculated as follows: MDA content = 6.45 (ΔOD532-600) − (0.56 OD450) and the final contents were expressed as µmol g^−1^ FW. To measure the electrolyte leakage percentage at respective growth stages of aromatic rice plants, the fresh leaf samples were thoroughly washed with deionized water and cut into small pieces. Leaf discs (0.3 g) were then placed in 10 mL deionized water and incubated for 6 h at 25 °C and electrical conductivity (EC) EC1 was recorded using an EC meter (SX-650, Sanxin, China). The leaf samples were again incubated for 2 h at 90 °C to record EC2. The electrolyte leakage (EL) percentage in leaf tissues of aromatic rice plants was estimated as: EL (%) = (EC1/EC2 × 100) [23].

### 2.6. Measurement of Enzymatic and Non-Enzymatic Antioxidant

In order to estimate antioxidant enzyme assay, fresh leaf samples (0.3 g) were crushed in liquid nitrogen and homogenized in 6 mL of 50 mM sodium phosphate buffer (pH 7.8) with a mortar and pestle in an ice bath. The homogenate was centrifuged (12,000× *g*, 10 min) to separate the supernatant from crude fibers and the aliquot of the supernatant was used to measure the activities of catalase, superoxide dismutase, peroxidase, and ascorbate peroxidase in aromatic rice leaves according to the methods described in a previous study [24]. The contents of non-enzymatic antioxidants (GSH and GSSG) at different growth stages of aromatic rice plants were determined by ‘A006-2-1’ and ‘A061-2-1’ kits, respectively, bought from Nanjing Jiancheng Bioengineering Institute (www.njjcbio.com (accessed on 3 September 2020)), China.

### 2.7. Total RNA Extraction and qRT-PCR Analysis

Fresh aromatic rice leaves were sampled at three prominent growth stages for total RNA extraction. Total RNA was extracted using TRIzol reagent (Invitrogen, Carlsbad, CA, USA). However, subsequent procedures for assessing the quality and quantity of total RNA, cDNA synthesizing, and Real Time PCR setting up were carefully adopted as described in our previous experiment [25]. Data on nucleotides sequence and specific annealing temperature can be found in Table S2. Three biological replicates of each sample were used, and by normalizing the Ct value relative to the ACTIN Ct value, the expression levels for each gene were calculated according to the 2-ΔΔCt method for quantification [26].

### 2.8. Estimation of Molybdenum and Cadmium Concentration in Different Plant Parts

The aromatic rice plant leaves were sampled at three prominent growth stages and ears (at reproductive stage) and grains (at maturity stage) and oven dried for estimating Mo and Cd concentrations. The plant samples (0.3 g) were digested in 5:1 (*v*/*v*) HNO_3_:HClO_4_ (5 mL) in a microwave oven (MLS 1200, Milestone, FKV, Italy). Mo and Cd concentrations were measured by ICP-mass spectrometry (ICP-MS) (ELAN DRC-e, PerkinElmer SCIEX, DE) and Atomic Absorption Spectrophotometer (AAS) (AA6300C, Shimadzu, Japan) [2,27].

### 2.9. Estimation of 2-acetyl-1-pyrroline (2AP) Content

Freshly collected grains were homogenized with dichloromethane and sodium sulfate for 2AP measurement. The 2AP content in aromatic rice grains was measured in combination with the Gas Chromatograph Mass Spectrometer (GCMS-QP 2010 Plus, Shimadzu Corporation, Japan) by the synchronization distillation and extraction (SDE) method. The measuring conditions were as follows: the gas chromatograph fitted with silica capillary column (30 m × 0.32 mm × 0.25 μm) of RTX-5MS (Shimadzu, Japan) and highly pure helium gas (carrier gas) with a flow rate of 2.0 mL min^−1^ (99.99%, Guangzhou Gases Co., Ltd., Guangzhou, China) [28,29].

### 2.10. Measurement of Grain Yield, Yield Attributes, and Grain Quality Characters of Aromatic Rice

The grain yield and yield contributing attributes of aromatic rice cultivar ‘Xiangyaxiangzhan’ were measured at the harvesting time. The total numbers of tillers pot^−1^ and numbers of productive tillers pot^−1^ were counted manually. One thousand grains were counted and weighed on the digital electric balance to achieve a 1000-grain weight (g), while the overall paddy weight obtained from each pot was calculated as grain yield pot^−1^. The grain quality features of aromatic rice ‘Xiangyaxiangzhan’ cultivar were determined in the harvested grains. The brown rice was obtained by a rice huller (Jiangsu, China) and the percentage of brown rice was determined as: (weight of brown rice/paddy weight × 100). For measuring brown rice milling percentage, a Jingmi testing rice miller (Zhejiang, China) was used and the percentage was calculated as: (weight of milled rice/original weight × 100). The milling degree percentage was determined as: (weight of milled rice/weight of brown rice × 100). The percentages of chalkiness degree and chalkiness rate were calculated via an SDE-A light box (Guangzhou, China). The head rice rate percentage was determined by separating the whole milled grains out of 100 grains. Infratec 1241 (FOSS-TECATOR) was used to determine the protein and moisture content percentage in aromatic rice grains.

### 2.11. Statistical Analysis

The data collected were statistically analyzed through two-way ANOVA within a particular growth stage using ‘Statistix 8.1’ (Analytical Software, Tallahassee, FL, USA). Mean variances were separated by an LSD test at *p* < 0.05. SigmaPlot 10.0 was used for graphical representations. The heat-map hierarchical analysis between treatments and different studied parameters was conducted by R 3.5.1.

## 3. Results

### 3.1. Effect of Mo Supply on Photosynthetic Pigments and Photosynthetic Efficiency and Chloroplast Configuration of Aromatic Rice Plants under Cd Toxicity

**Figure 1:**
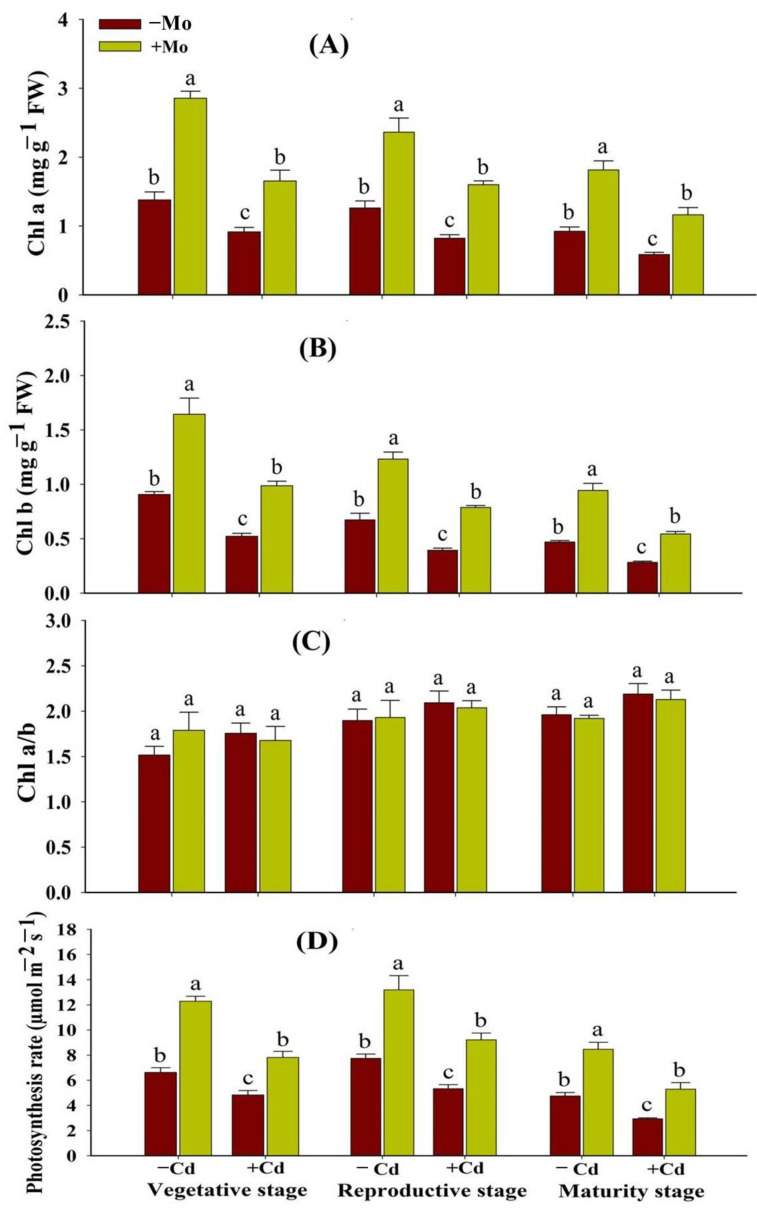
Effect of Mo supply and Cd toxicity on Chl a (**A**), Chl b (**B**), ratio of Chl a/b (**C**), and photosynthetic rate (P_n_) (**D**), in leaves of aromatic rice ‘Xiangyaxiangzhan’ cultivar, at the vegetative, reproductive, and maturity stages. Bars above means indicate ±S.E. of four independent replicates (*n* = 4) and different small alphabetical letters above means reveal significant differences among treatments within a particular growth stage according to the LSD test (*p* < 0.05).

In the absence of Mo, Cd stress significantly (*p* < 0.05) decreased the photosynthetic pigments in leaves of aromatic rice plants at different growth stages; however, Mo supply enhanced the chlorophyll contents under −/+ Cd toxicity (Figure 1A,B). Under Cd stress, Mo supply enhanced chlorophyll a content by 80.88%, 94.37%, and 88.33%, and chlorophyll b content by 89.12%, 99.32%, and 92.27% at vegetative, reproductive, and maturity stages, respectively, as compared to −Mo treatments (Figure 1). Similarly, Cd toxicity significantly reduced the photosynthetic efficiency at different growth stages while Mo application enhanced the photosynthetic rate of aromatic rice plants. Under Cd stress, Mo application enhanced the photosynthetic rate by 61.67%, 72.79%, and 79.66% at the vegetative, reproductive, and maturity stages, respectively (Figure 1D).

**Figure 2:**
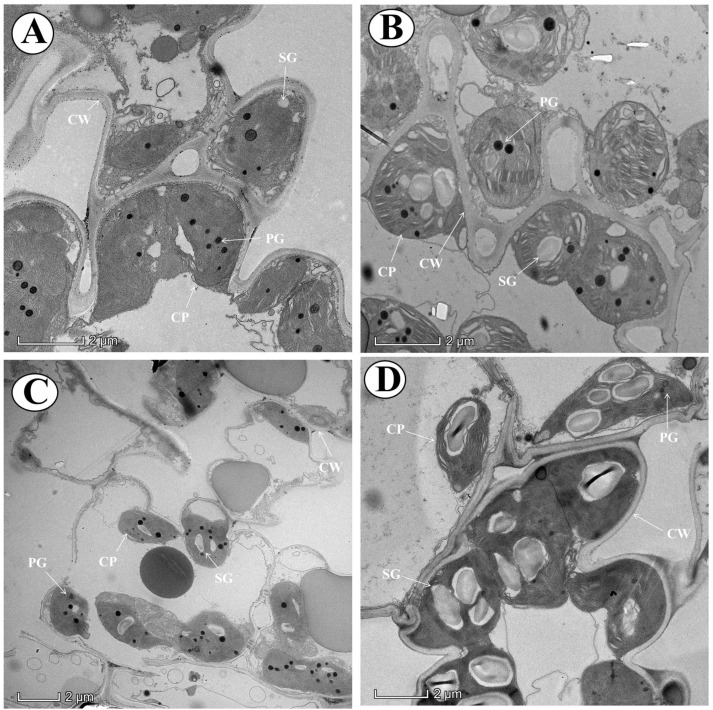
Effect of Mo supply and Cd toxicity on the transmission electron microscopy (TEM) images of aromatic rice ‘Xiangyaxiangzhan’ cultivar. Aromatic rice plants were treated with (**A**) (0 and 0 mg), (**B**) (0.15 and 0 mg), (**C**) (0 and 100 mg), and (**D**) (0.15 and 100 mg) of Mo and Cd, respectively. CP, chloroplasts; PG, plastoglobuli; CW, cell wall; SG, starch grains.

The current study revealed that Cd toxicity severely affected the cell ultra-structural configurations and the integrity of chloroplasts in aromatic rice plants (Figure 2). However, under Mo-supplied treatments, the chloroplasts were observed to be comparatively regularly oval shaped, more consistent, and with well-organized arrangement and accumulated greater starch grains (Figure 2).

### 3.2. Influence of Mo Supply on Osmo-Regulation under Cd Toxicity

**Figure 3:**
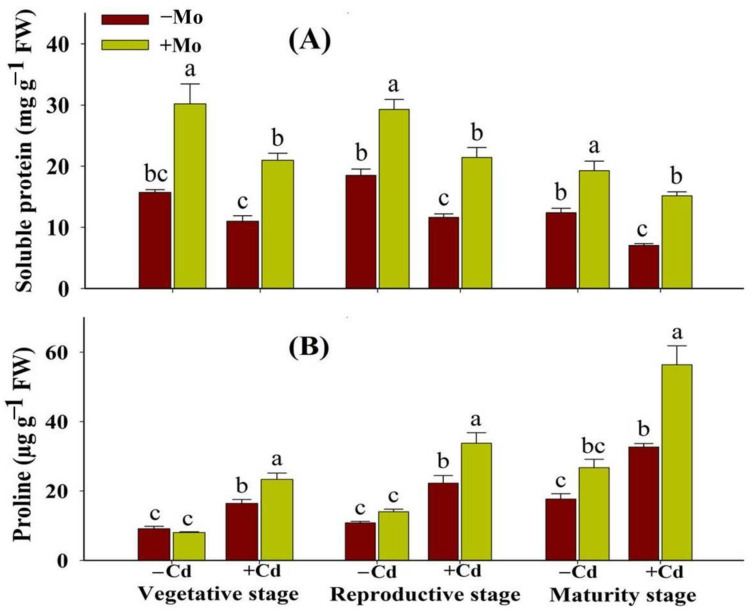
Effect of Mo supply and Cd toxicity on soluble protein (**A**) and proline contents (**B**) in leaves of aromatic rice ‘Xiangyaxiangzhan’ cultivar, at the vegetative, reproductive, and maturity stages. Bars above means indicate ±S.E. of four independent replicates (*n* = 4) and different small alphabetical letters above means reveal significant differences among treatments within a particular growth stage according to the LSD test (*p* < 0.05).

Our findings showed that Cd stress significantly decreased the soluble protein content in aromatic rice at different crop growth stages; however, such reductions were more severe at maturity stage (Figure 3A). However, Mo supply enhanced the soluble protein content with/without Cd toxicity compared to −Mo treatment. Under Cd stress, Mo supply enhanced the soluble protein content by 89.30%, 84.15%, and 115.37% at the vegetative, reproductive, and maturity stages, respectively, compared with −Mo treatment (Figure 3A). In contrast to soluble protein, proline content was significantly enhanced under Cd stress at different growth stages compared to without Cd toxicity (Figure 3B). Moreover, under Cd stress, Mo supply further enhanced the proline contents by 41.96%, 51.95%, and 72.54% at the vegetative, reproductive, and maturity stages, respectively, relative to −Mo treatments (Figure 3B).

### 3.3. Effect of Mo Supply on Membrane Integrity in Aromatic Rice under Cd Stress

**Figure 4:**
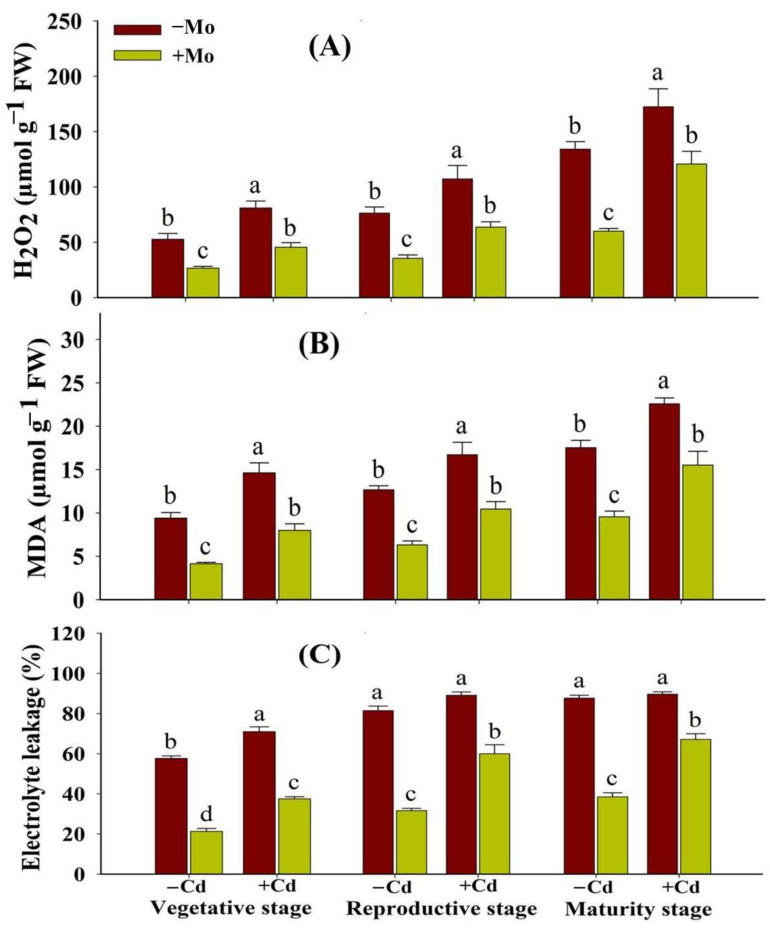
Effect of Mo supply and Cd toxicity on hydrogen peroxide (H_2_O_2_) content (**A**), malondialdehyde (MDA) (B), and electrolyte leakage (EL) (**C**), in leaves of aromatic rice ‘Xiangyaxiangzhan’ cultivar, at the vegetative, reproductive, and maturity stages. Bars above means indicate ±S.E. of four independent replicates (*n* = 4) and different small alphabetical letters above means reveal significant differences among treatments within a particular growth stage according to the LSD test (*p* < 0.05).

The lipid peroxidation (MDA production), electrolyte leakage (EL), and H_2_O_2_ accumulations are associated with the membrane integrity. The results indicated that Cd toxicity considerably increased the levels of H_2_O_2_, MDA, and EL in leaves of aromatic rice plants (Figure 4). However, Mo application reduced H_2_O_2_, MDA, and EL in leaf tissues of aromatic rice plants. Under Cd stress, Mo supply reduced the oxidative damage by lowering the levels of H_2_O_2_ (43.81%, 40.55%, and 29.95%), MDA (45.43%, 37.48%, and 31.24%), and EL (47.15%, 32.71%, and 25.14%) at the vegetative, reproductive, and maturity stages, respectively, compared with −Mo treatment (Figure 4).

### 3.4. Effect of Mo Supply on the Activities and Transcript Abundance of Enzymatic Antioxidants under Cd Toxicity in Aromatic Rice

**Figure 5:**
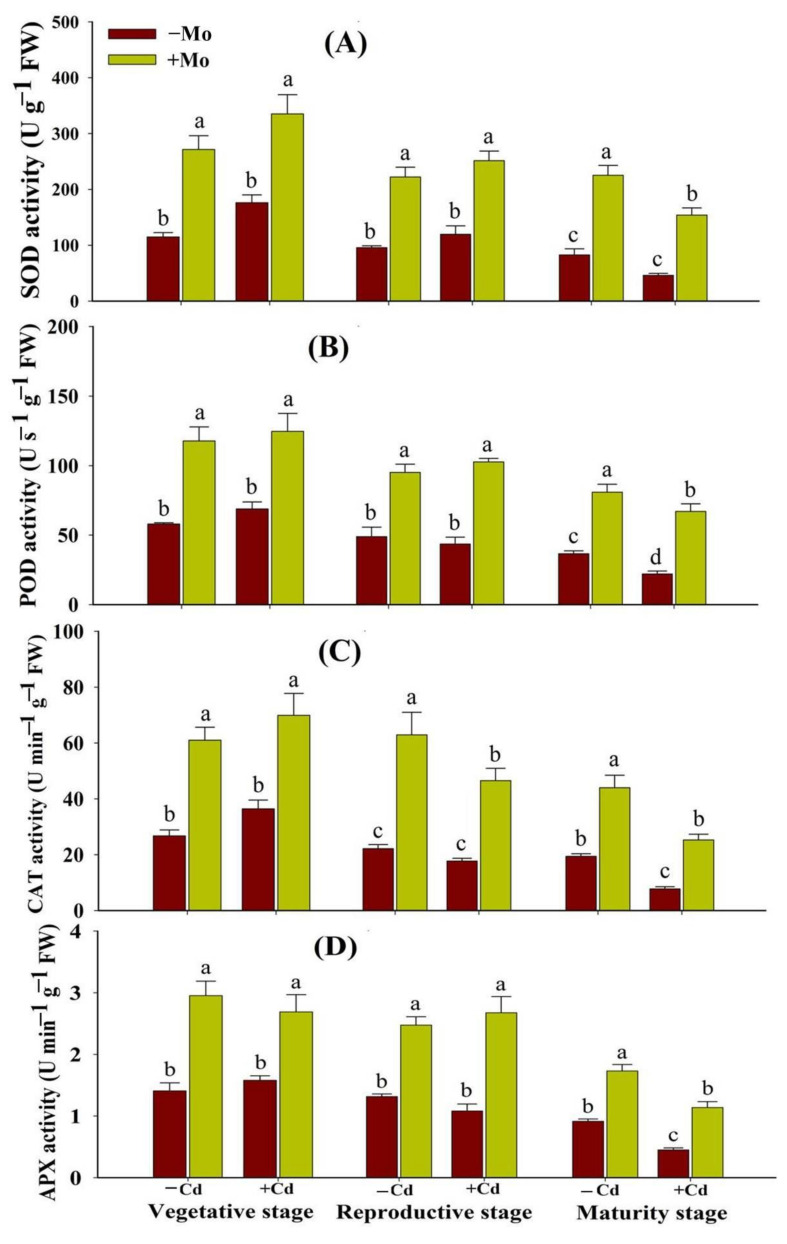
Effect of Mo supply and Cd toxicity on the activities of enzymatic antioxidants: superoxide dismutase (SOD) (**A**); peroxidase (POD) (**B**); catalase (CAT) (**C**); and ascorbate peroxidase (APX) (**D**), in the leaves of aromatic rice ‘Xiangyaxiangzhan’ cultivar, at the vegetative, reproductive, and maturity stages. Bars above means indicate ±S.E. of four independent replicates (*n* = 4) and different small alphabetical letters above means reveal significant differences among treatments within a particular growth stage according to the LSD test (*p* < 0.05).

**Figure 6:**
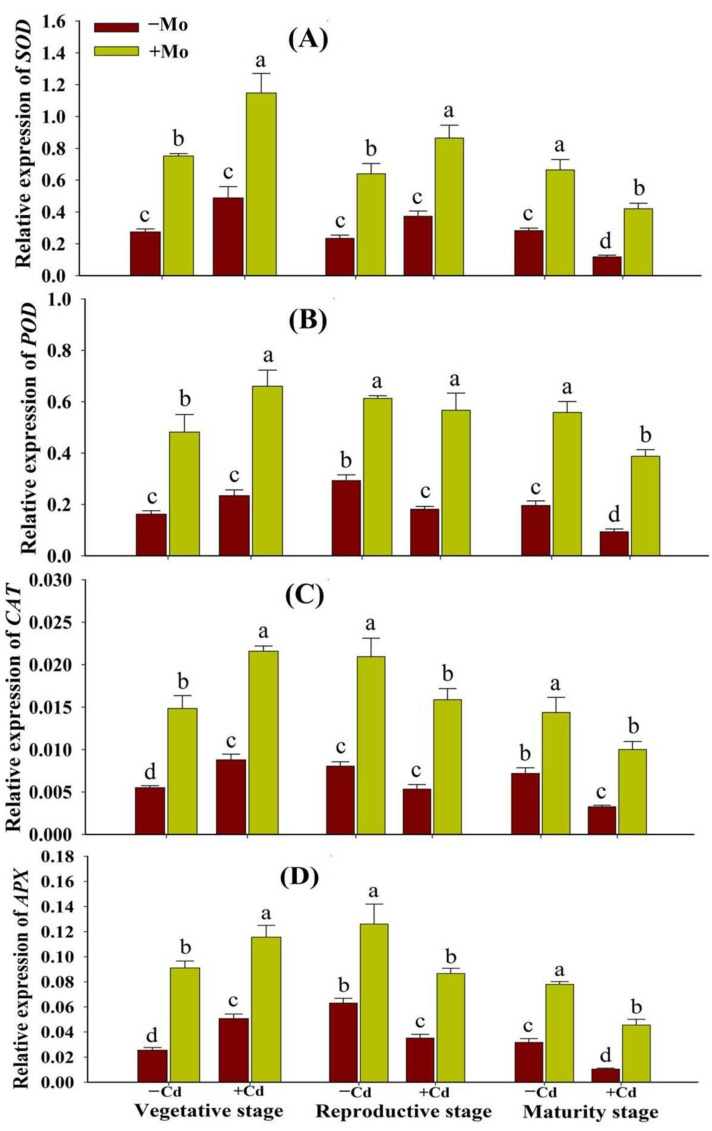
Effect of Mo supply and Cd toxicity on qRT-PCR analysis of antioxidant enzyme-related transcripts of superoxide dismutase (SOD) (**A**); peroxidase (POD) (**B**); catalase (CAT) (**C**); and ascorbate peroxidase (APX) (**D**), in the leaves of aromatic rice ‘Xiangyaxiangzhan’ cultivar, at the vegetative, reproductive, and maturity stages. Bars above means indicate ±S.E. of four independent replicates (*n* = 4) and different small alphabetical letters above means reveal significant differences among treatments within a particular growth stage according to the LSD test (*p* < 0.05).

Under Cd stress, the antioxidant enzyme activities and their respective transcript levels were higher at the vegetative stage while being decreased at the maturity stage. The Mo supply significantly enhanced the enzymatic antioxidant activities and transcript abundance at different growth stages (vegetative, reproductive, and maturity) (Figure 5). Mo application regulated the activities of SOD by 90.29%, 109.99%, and 233.30%; POD by 80.96%, 135.73%, and 204.01%; CAT by 91.69%, 162.42%, and 225.60%; and APX by 70.09%, 147.42%, and 153.48% under Cd toxicity, at the vegetative, reproductive, and maturity stages, respectively, compared with −Mo treatment (Figure 5). Similarly, Mo application increased the expression levels of SOD, POD, CAT, and APX genes in the leaf tissues of aromatic rice plants with/without Cd stress at different growth stages (Figure 6), indicating that Mo supply strengthened the antioxidant defense system of aromatic rice plants through increasing antioxidant enzyme activities and expressions under stress conditions.

### 3.5. Influence of Mo Supply on Non-Enzymatic Antioxidants in Aromatic Rice under Cd-Stress

**Figure 7:**
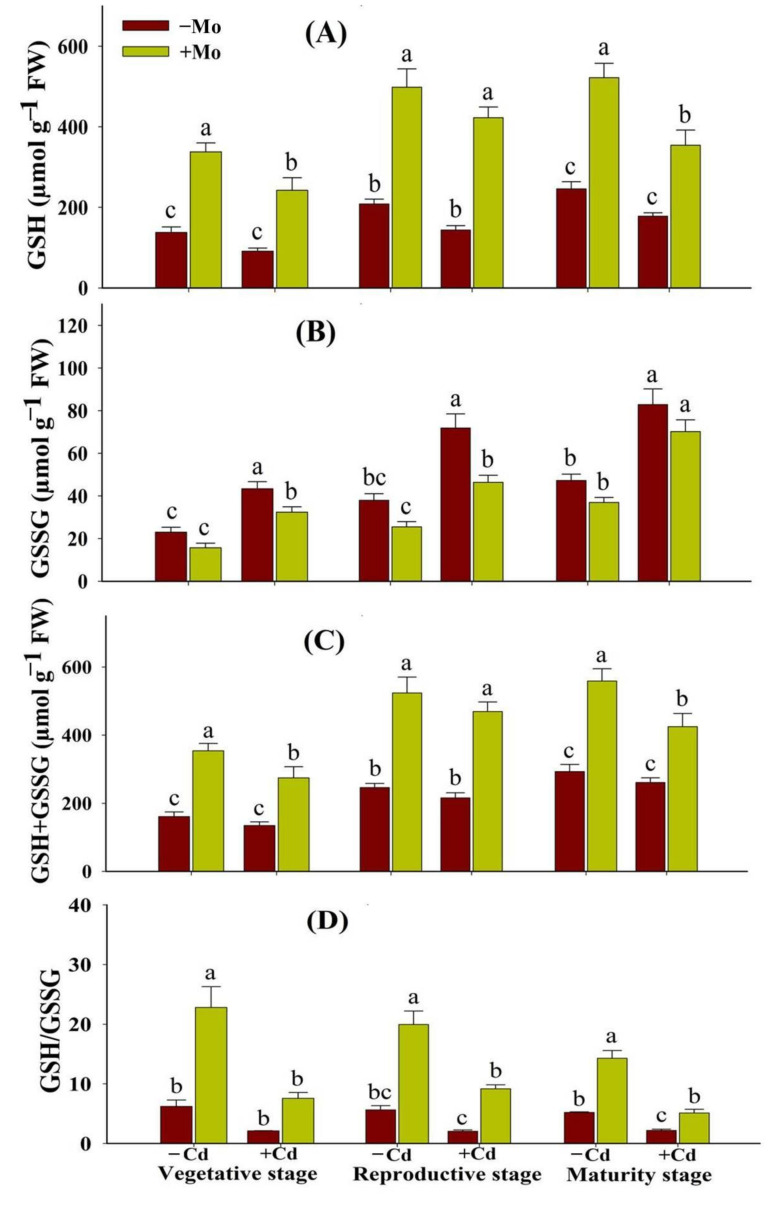
Effect of Mo supply and Cd toxicity on reduced glutathione (GSH) (**A**); oxidized glutathione (GSSG) (**B**); total glutathione (GSSG + GSH) (**C**); and ratio of GSH/GSSG (**D**), in the leaves of aromatic rice ‘Xiangyaxiangzhan’ cultivar, at the vegetative, reproductive, and maturity stages. Bars above means indicate ±S.E. of four independent replicates (*n* = 4) and different small alphabetical letters above means reveal significant differences among treatments within a particular growth stage according to the LSD test (*p* < 0.05).

The Cd stress reduced the GSH and total glutathione contents while significantly increasing GSSG concentration at different growth stages of aromatic rice (Figure 7). However, Mo supply enhanced the GSH (165.82%, 193.56%, and 99.11%) and GSH + GSSG (104.12%, 117.27%, and 62.77%) levels while decreasing GSSG (25.39%, 35.43%, and 15.29%) contents under Cd stress, at the vegetative, reproductive, and maturity stages, respectively (Figure 7A–C). Moreover, Mo supply increased the GSH/GSSG in leaves at prominent growth stages of aromatic rice plants (Figure 7D).

### 3.6. Mo and Cd Concentrations in Different Plant Parts of Aromatic Rice Plants

The results revealed that Mo concentrations in leaves, ears, and grains of aromatic rice plants were significantly increased with Mo supply; however, non-significant effects were observed in Mo concentration under −/+ Cd stress in various plant parts of aromatic rice (Table S3). The Cd concentrations were increased in leaves, ears, and grains of aromatic rice plants under Cd stress. However, Mo application decreased the Cd concentration in leaves by 32.51%, 36.40%, and 24.82% at the vegetative, reproductive, and maturity stages, respectively, and 25.35% in ears (at reproductive stage) and 29.75% in grains (at maturity stage) under Cd toxicity (Table S3), suggesting that Mo supply inhibited the Cd accumulation and consequently mitigated the Cd toxicity through the least accretion in the edible part (grains) of aromatic rice.

### 3.7. Influence of Mo Supply on Yield Traits and Grain Quality Characteristics of Aromatic Rice under Cd Stress

Pronounced variations in the growth of aromatic rice plants were observed under the influence of Mo supply and Cd toxicity treatments (Figure S1). The Cd stress caused considerable reductions in grain yield and yield contributing traits and also deteriorated the grain quality characteristics of aromatic rice. However, Mo supply mitigated Cd-provoked adversities in aromatic rice and significantly improved grain yield and associated attributes (except 1000-grain weight) under Cd toxicity (Table 1). Compared with −Mo treatment, Mo supply improved the total number of tillers pot^−1^ (61.04%), productive tillers pot^−1^ (69.86%), filled grain percentage (25.07%), 1000-grain weight (10.15%), grains panicle^−1^ (40.07%), and grain yield pot^−1^ (64.75%) of aromatic rice under Cd stress (Table 1).

Rice quality attributes including 2AP contents, brown rice rate, milled rice rate, head rice rate, and protein and moisture contents were considerably reduced while chalkiness rate and chalkiness degree increased under Cd toxicity, indicating that Cd stress deteriorated aromatic rice quality. However, Mo supply restored the grain quality characters under Cd toxicity (Table 2) and improved aromatic rice quality under Cd stress. Compared with −Mo treatment, Mo supply increased the 2AP contents (77.09%), milling degree (14.24%), milled rice rate (12.35%), head rice rate (25.74%), protein contents (29.35%), brown rice rate (5.59%), and moisture content (2.67%), while reducing the chalkiness degree (26.08%) and chalkiness rate (22.71%) in aromatic rice grains under Cd stress (Table 2).

### 3.8. Relationships

**Figure 8:**
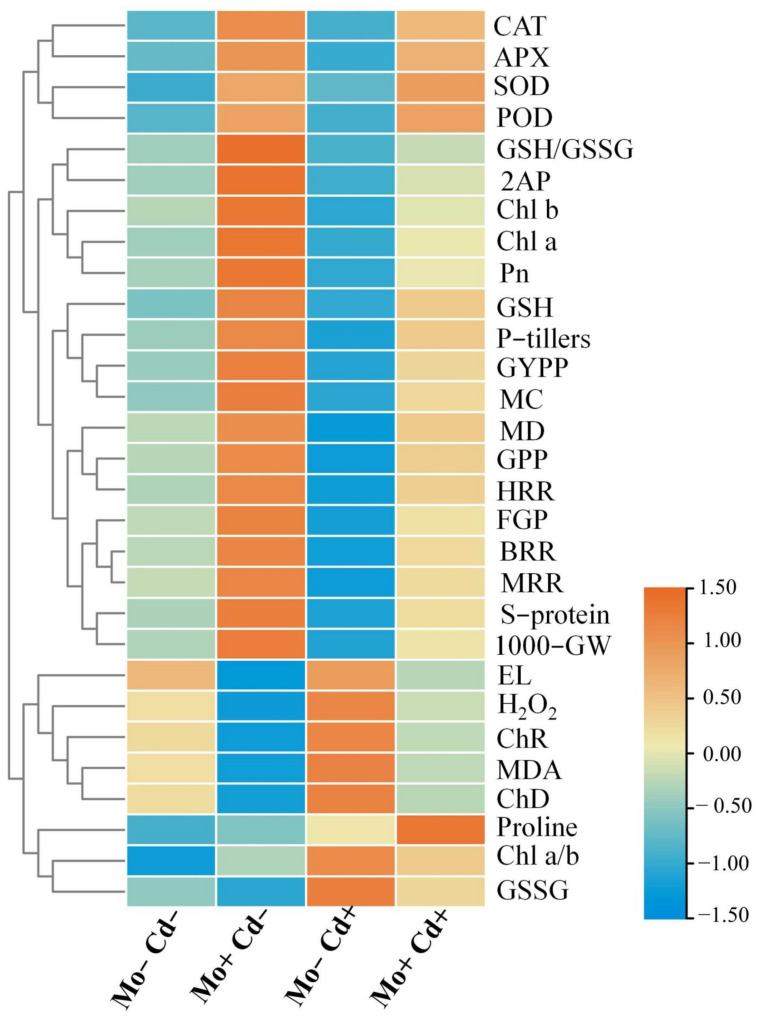
Heat-map reveals a hierarchical clustering analysis between different treatments and studied parameters of aromatic rice plants. Abbreviated names are as follows: 2AP (2-acetyl-1-pyrroline), SOD (superoxide dismutase activity), POD (peroxide activity), CAT (catalase activity), APX (ascorbate peroxidase activity), Chl a (chlorophyll a), Chl b (chlorophyll b), Pn (photosynthetic rate), GSH (reduced glutathione), GSSG (oxidized glutathione), P-tillers (productive tillers), HRR (head rice rate), MRR (milled rice rate), FGP (filled grain percentage), BRR (brown rice rate), S-protein (soluble protein), 1000-GW (1000-grain weight), EL (electrolyte leakage), H_2_O_2_ (hydrogen peroxide), ChR (chalkiness rate), ChD (chalkiness degree), MDA (malondialdehyde contents), MC (moisture content), MD (milling degree), GPP (grains per panicle), GYPP (grain yield per pot).

The hierarchical analysis revealed that Cd toxicity negatively affected the growth parameters, yield attributes, and grain quality characteristics of aromatic rice plants and concomitantly these inhibitory effects are correlated with the reduced photosynthetic pigments and weakened enzymatic and non-enzymatic antioxidant defense mechanism while triggering oxidative damage. However, Mo supply established strongly positive correlations with the grain yield attributes, quality characters, strengthening photosynthetic apparatus, and antioxidant defense system, and reduced the oxidative damage. In essence, this hierarchical correlation analysis reveals that Mo supply played an efficient role in mitigating the Cd-induced toxicity effects on the plant growth, grain yield, and yield-contributing attributes, and quality characteristics of aromatic rice (Figure 8).

## 4. Discussion

All heavy metals, particularly Cd toxicity, inevitably provoke perturbations in various physio-biochemical processes, photosynthetic apparatus, plant metabolism, and antioxidant protection mechanisms, thereby resulting in significant yield reduction and quality deterioration in crop plants [4,30,31,32]. Thus, Cd stress-alleviating approaches in plant growth and developmental cycles, yield reductions, and quality deteriorations through strengthened plant metabolism continue to be challenging goals for plant scientists. Molybdenum (Mo), an essential and anti-stress micronutrient, has garnered substantial consideration due to its primary involvement in multiple plant growth and production processes and enhancing oxidative stress resistance under cold, drought, and heavy metal toxicities [13,15,24]. The current experiment established a number of mitigating roles of Mo supply on photosynthetic apparatus, plant metabolism, antioxidant defense system, yield attributes, and quality characteristics of aromatic rice cultivar ‘Xiangyaxiangzhan’ under Cd toxicity.

Our results revealed that Cd toxicity significantly reduced the photosynthetic pigments and photosynthetic efficiency at the vegetative, reproductive, and maturity stages of aromatic rice plants (Figure 1), which is in line with the results of several previous reports, who documented the Cd-induced reduction in chlorophyll contents and photosynthetic efficiency [33,34]. The Mo application was found to increase the chlorophyll contents and photosynthetic efficiency in Cd-stressed aromatic rice plants (Figure 1), which might be ascribed to Mo-induced reductions in oxidative damage and maintenance of chloroplast ultra-structure (Figure 2 and Figure 4) [35]. Similarly, previous studies reported that Mo supply increased the leaf gaseous exchange attributes, photosynthetic pigments, and chloroplast integrity and configurations in Triticum aestivum [36] and Fragaria ananassa [37].

Biosynthesis and relative accumulation of compatible solutes/osmolytes are well-recognized due to a wide range of their functions, i.e., protection and stabilization of cellular membranes, protection of several enzymes, acting as osmoticum for turgor maintenance, and scavenging roles against ROS (e.g., proline) in plants [38]. In our study, Cd toxicity reduced soluble protein contents, which might be due to greater oxidative damage, and these results are concomitant with previous studies that Cd toxicity stimulated soluble protein degradation through higher protease activity [39] and unnecessary ROS generation [40]. The Mo supply was found to enhance the levels of both soluble protein and proline contents at different growth stages of aromatic rice plants under Cd stress (Figure 3), suggesting the ameliorative role of Mo in improving and maintaining higher plant osmotic balance under stress conditions.

The Cd toxicity causes overproduction of free radicals and ROS, causing ultra-structural and functional alterations in cell nuclei, DNA, lipids, and proteins. The present study revealed that Cd stress stimulated the oxidative stress as evident from higher production of H_2_O_2_, MDA, and loss of membrane integrity (greater EL) in aromatic rice plants (Figure 4). However, Mo supply significantly reduced MDA, H_2_O_2_, and EL in leaf tissues at prominent growth stages of aromatic rice plants (Figure 4), suggesting that Mo supply mitigated Cd-provoked intracellular membrane disruptions throughout the growth period of aromatic rice plants. These Mo-induced mitigating approaches to preserve and retain bilayer membranes and safeguard cell membranes from oxidative stress destructions have also been reported in strawberry [41].

Under stress conditions, both enzymatic and non-enzymatic antioxidants play key roles to scavenge the ROS and counteract the oxidative damage in plants. In the present study, Cd toxicity aggravated oxidative damage in aromatic rice plants, while Mo application alleviated oxidative stress, which could be ascribed to the enhancement of antioxidant enzyme activities and expressions (Figure 5 and Figure 6). The protection of plants by antioxidant defense systems against oxidative damage was triggered by antioxidant enzymes including SOD, CAT, APX, and POD [42,43,44]. Generally, ROS is scavenged by antioxidant enzymes. For example, SOD catalyzes O_2_^-^ into H_2_O_2_, and then H_2_O_2_ is disintegrated by CAT and APX [42,45,46]. Previous studies also reported that Mo supply relieved oxidative damage by improving the antioxidant defense ability of plants under cold, drought, salt stress, and heavy metals [11,13,47]. However, the mechanisms by which Mo improves antioxidant defense ability remain to be investigated. The non-enzymatic antioxidants (GSH and GSSG) also serve as a redox buffer under various heavy metals [48]. GSH shields guard cells from oxidative destruction and plays a major role in reducing the bulk of ROS. However, under adverse conditions as cells undergo greater oxidative stress damages, GSSG accumulates and the GSH to GSSG ratio decreases. Thus, estimating the GSH to GSSG ratio (GSH/GSSG) is a valuable indicator for assessing the rate of oxidative stress in cells and tissues [49]. In this study, under Cd toxicity, Mo supply maintained a higher GSH/GSSG ratio in leaves of aromatic rice plants at the vegetative, reproductive, and maturity stages (Figure 7D), demonstrating that Mo supply mitigated the Cd-provoked stress adversities in aromatic rice. These results thus indicate that Mo supply has improved both enzymatic and non-enzymatic antioxidants during the growth cycle of aromatic rice plants, i.e., the vegetative, reproductive, and maturity stages and mitigated Cd-induced toxicity effects on plant physio-biochemical processes as is evident from significant reductions in ROS production, MDA contents, and electrolyte leakage under Cd-stressed treatments.

In rice, grain yield depends upon productive tillers, sterility percentage, number of grains panicle-1, and 1000-grain weight, while 2AP content, milling recovery, and chalkiness rate are important indices for quality estimation of aromatic rice [2,28]. In the present study, Cd toxicity reduced the yield-contributing attributes, i.e., productive tillers per pot, number of grains per panicle, and filled grain percentage (Table 1), and deteriorated the rice quality traits through reduced 2AP contents, brown rice rate, milled rice rate, milling degree, head rice rate, protein contents, while increasing chalkiness rate and chalkiness degree (Table 2), indicating that Cd toxicity reduced aromatic rice yield and deteriorated quality traits through all the contributing traits being affected. Previous studies have also reported significant reductions in yield and quality traits under Cd toxicity in different aromatic rice cultivars [8,17]. Similarly, our findings agree with previous studies reporting that Cd toxicity resulted in yield reductions and quality deterioration through a brutally impeded plant metabolism and photosynthetic system, and higher Cd concentration in plant parts and stimulated oxidative stress [4,15]. However, Mo supply substantially increased grain yield and quality traits of aromatic rice under Cd stress. The probable explanation is that Mo supply mitigated Cd-induced inhibitions on plant metabolism through strengthening the enzymatic and non-enzymatic antioxidant defense system, fortifying photosynthetic apparatus, and inhibiting the absorption and translocation of Cd in aromatic rice plants.

Taken together, our findings infer that Cd toxicity hampered aromatic rice plant growth, yield attributes, and grain quality traits through destabilizing plant metabolism, increasing oxidative damage, undermining the plant protection system, and distressing the photosynthetic apparatus. However, Mo supply alleviated the Cd-induced inhibitions on plant metabolism and improved aromatic rice yield and grain quality traits by strengthening the photosynthetic system and antioxidant protection mechanisms. Future studies can, however, be meditated to examine the possible role/s of Mo supply during 2AP biosynthesis pathways in different aromatic rice cultivars under heavy metal-polluted soils.

## 5. Conclusions

The current experiment revealed that Cd stress hampered the plant growth, grain yield, and quality traits of aromatic rice. The Cd toxicity triggered the production of H_2_O_2_ and electrolyte leakage, presumably by desynchronizing the ROS-scavenging mechanism. Nevertheless, Mo supply proficiently relieved Cd-provoked inhibitory effects on the plant metabolism, grain yield, and quality characteristics of aromatic rice, which could mainly be ascribed to reduced Cd uptake, reinforced photosynthetic apparatus, consistent chloroplast ultra-structure, and a higher scavenging ROS amount. Thus, our findings concluded that Mo supply mitigated the Cd-provoked inhibitory effects on plant growth, physio-biochemical processes, and antioxidant defense system during the plant growth cycle (vegetative, reproductive, and maturity stages), thereby enhancing grain yield and quality traits of aromatic rice.

## Figures and Tables

**Table 1:** Influence of Mo supply and Cd toxicity on aromatic rice yield and associated attributes.

Cd Toxicity	Mo Application	Tillers Pot^−1^	Productive Tillers Pot^−1^	Grains Panicle^−1^	Filled Grain Percentage	1000-Grain Weight (g)	Grain Yield Pot^−1^ (g)
−Cd	−Mo	26.97 ± 1.36 ^b^	21.31 ± 1.48 ^c^	128.91 ± 4.59 ^b^	68.24 ± 2.23 ^b^	18.11 ± 0.42 ^b^	46.11 ± 2.47 ^c^
	+Mo	40.08 ± 2.70 ^a^	32.46 ± 1.47 ^a^	165.21 ± 8.61 ^a^	83.35 ± 5.15 ^a^	20.30 ± 0.85 ^a^	73.36 ± 4.32 ^a^
+Cd	−Mo	19.89 ± 0.61 ^c^	16.02 ± 1.40 ^d^	103.72 ± 3.48 ^c^	57.76 ± 2.81 ^c^	16.97 ± 0.67 ^b^	35.26 ± 1.43 ^d^
	+Mo	32.05 ± 1.59 ^b^	27.20 ± 1.18 ^b^	145.28 ± 5.67 ^b^	72.25 ± 1.88 ^b^	18.70 ± 0.64 ^ab^	58.08 ± 4.19 ^b^
LSD _(*p* < 0.05)_		5.35	4.27	18.20	10.09	2.05	10.26

−Mo and +Mo denote 0 and 0.15 mg of molybdenum supplied as ammonium molybdate [(NH_4_)_6_Mo_7_O_24_·4H_2_O]), whilst −Cd and +Cd denote 0 and 100 mg of cadmium supplied as cadmium chloride (CdCl_2_·2.5H_2_O) kg^−1^ soil, respectively. The numeric values reflect means from four independent replicates (±S.E.) with different treatments. Data presented in individual columns indicated with dissimilar letters differ significantly by LSD test at *p* < 0.05.

**Table 2:** Influence of Mo supply and Cd toxicity on aromatic rice grain quality traits.

Cd Toxicity	Mo Application	Brown Rice Rate (%)	Milled Rice Rate (%)	Milling Degree (%)	Head Rice Rate (%)	Chalkiness Rate (%)	Chalkiness Degree (%)	Moisture Content (%)	Protein Content (%)	2AP Content (ng g^−1^ FW)
−Cd	−Mo	73.89 ± 3.78 ^a^	60.14 ± 1.52 ^bc^	77.91 ± 1.59 ^b^	56.89 ± 3.82 ^bc^	33.66 ± 2.44 ^b^	16.94 ± 0.53 ^b^	12.27 ± 0.08 ^b^	6.47 ± 0.14 ^c^	144.35 ± 7.40 ^b^
	+Mo	77.81 ± 1.87 ^a^	66.42 ± 1.70 ^a^	85.83 ± 1.46 ^a^	68.53 ± 5.26 ^a^	24.06 ± 1.28 ^c^	11.75 ± 0.58 ^c^	12.68 ± 0.18 ^a^	8.49 ± 0.22 ^a^	297.08 ± 23.91 ^a^
+Cd	−Mo	71.24 ± 1.78 ^a^	55.20 ± 1.71 ^c^	71.55 ± 1.82 ^c^	49.57 ± 2.04 ^c^	39.78 ± 1.70 ^a^	20.62 ± 0.94 ^a^	12.12 ± 0.13 ^b^	5.50 ± 0.09 ^d^	97.01 ± 6.39 ^c^
	+Mo	75.22 ± 2.31 ^a^	62.02 ± 2.35 ^ab^	81.74 ± 2.05 ^ab^	62.34 ± 2.94 ^ab^	30.74 ± 1.71 ^b^	15.25 ± 0.56 ^b^	12.45 ± 0.07 ^ab^	7.12 ± 0.12 ^b^	171.79 ± 14.58 ^b^
LSD _(*p* < 0.05)_		7.89	5.69	5.38	11.42	5.50	2.07	0.38	0.47	45.69

−Mo and +Mo denote 0 and 0.15 mg of molybdenum supplied as ammonium molybdate [(NH_4_)_6_Mo_7_O_24_·4H_2_O]), whilst −Cd and +Cd denote 0 and 100 mg of cadmium supplied as cadmium chloride (CdCl_2_·2.5H_2_O) kg^−1^ soil, respectively. The numeric values reflect means from four independent replicates (±S.E.) with different treatments. Data presented in individual columns indicated with dissimilar letters differ significantly by LSD test at *p* < 0.05.

## Data Availability

Data is contained within the article and Supplementary Materials.

## Supplementary Materials

The following are available online at https://www.mdpi.com/article/10.3390/antiox10060838/s1, Figure S1: Pictorial view of aromatic rice plants grown under molybdenum (Mo) and cadmium (Cd) treatments, Table S1: The experimental soil’s chemical properties, Table S2: Primer sequences used for qRT-PCR amplification, Table S3: Influence of molybdenum supplementation and cadmium toxicity on Mo and Cd concentration at different growth stages in leaves, ears and grains of aromatic rice.
