# Supplementary material for: Molybdenum-Induced Regulation of Antioxidant Defense-Mitigated Cadmium Stress in Aromatic Rice and Improved Crop Growth, Yield, and Quality Traits
Source: Antioxidants (Basel). 2021 May 24;10(6):838. doi: 10.3390/antiox10060838 (PMC8225192; doi:10.3390/antiox10060838)
Supplement: Supplementary file 1 [file antioxidants-10-00838-s001.zip › antioxidants-1192265-supplementary.pdf]

Supplementary Figure S1:

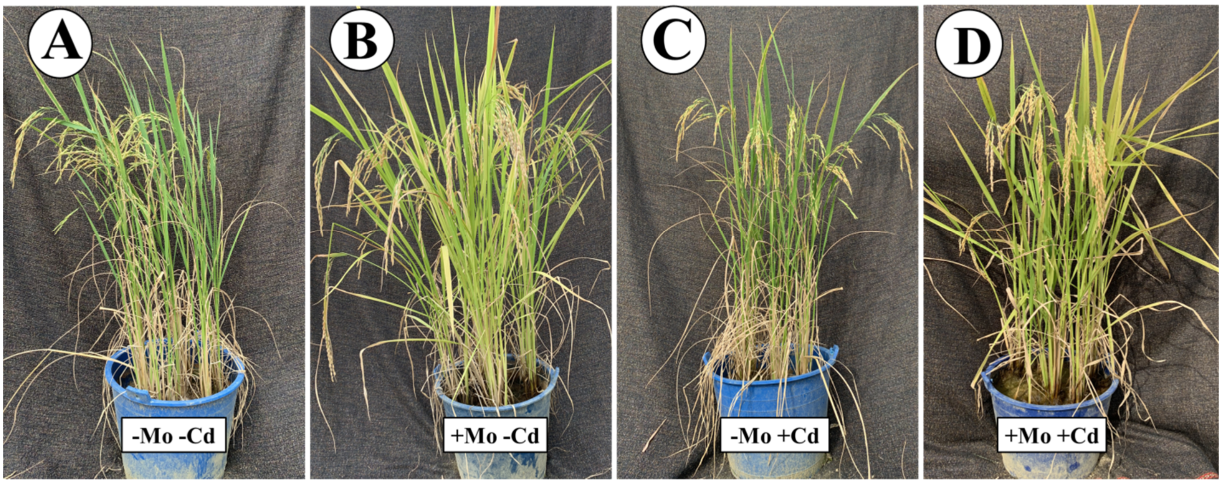

Pictorial view of aromatic rice plants grown under molybdenum (Mo) and cadmium (Cd) treatments. The treatment combinations represent as: -Mo-Cd: 0 and 0 mg, +Mo-Cd: 0.15 and 0 mg, -Mo+Cd: 0 and 100 mg, +Mo+Cd: 0.15 and 100 mg of Mo and Cd per kg of soil respectively. The molybdenum was supplied as ammonium molybdate [$(NH_4)6Mo_7O_{24}$•$4H_2O$] and Cd as $CdCl_2$•$2.5H_2O$ per kg of soil.

Supplementary Table S1: The experimental soil's chemical properties

Properties	Contents
O.M. (g kg^{-1})	20.32
Total N (g kg^{-1})	1.26
Total P (g kg^{-1})	0.87
Total K (g kg^{-1})	16.94
pH	5.61
Available N (mg kg^{-1})	85.28
Available P (mg kg^{-1})	9.25
Available K (mg kg^{-1})	118.39
Available Mo (mg kg^{-1})	0.072
Cd (mg kg^{-1})	2.13

Supplementary Table S2: Primer sequences used for qRT-PCR amplification

**Accession no.**	**Genes**	**Strand**	**primer sequences 5' to 3'**	**Annealing temperature (Tm)**
Os07g0694700	*APX*	F	TACGCCGACTTCTACCAGC	58 °C
		R	TTTATTACAACCGCCACGA	
Os06g51150	*CAT*	F	GCACAGTTTGACAGGGAG	56 °C
		R	GTCTTTGGACTTGGCTTG	
Os10g0109600	*POD*	F	CGACGATTTCTACGACTACAT	57 °C
		R	TGATTGAGGAGGTTCTGGT	
Os07g0665200	*SOD*	F	TGTCAACTGGACCACACTTC	55 °C
		R	ACTTAAAACGCATGCACTCA	
Os03g50885	*ACTIN*	F	TGCCAAGGCTGAGTACGACGA	57 °C
		R	CAAGCAGGAGGACGGCGATA	

Supplementary Table S3: Influence of molybdenum supplementation and cadmium toxicity on Mo and Cd concentration at different growth stages in leaves, ears and grains of aromatic rice

Growth stages	Cd toxicity	Mo supply	Cd concentration in leaves (μg g^{-1} DW)	Cd concentration in ears/grains (μg g^{-1} DW)	Mo concentration in leaves (μg g^{-1} DW)	Mo concentration in ears/grains (μg g^{-1} DW)
Vegetative stage	-Cd	-Mo	0.42 ± 0.04^{c}	-	0.095 ± 0.008^{c}	-
		+Mo	0.28 ± 0.02^{c}	-	0.220 ± 0.017^{b}	-
	+Cd	-Mo	11.41 ± 1.21^{a}	-	0.127 ± 0.006^{c}	-
		+Mo	7.70 ± 0.69^{b}	-	0.275 ± 0.020^{a}	-
Reproductive stage	-Cd	-Mo	0.49 ± 0.03^{c}	0.019 ± 0.001^{c}	0.134 ± 0.008^{b}	0.034 ± 0.003^{b}
		+Mo	0.30 ± 0.02^{c}	0.012 ± 0.001^{c}	0.266 ± 0.019^{a}	0.074 ± 0.005^{a}
	+Cd	-Mo	16.56 ± 1.91^{a}	0.546 ± 0.055^{a}	0.149 ± 0.016^{b}	0.041 ± 0.003^{b}
		+Mo	10.53 ± 0.95^{b}	0.408 ± 0.027^{b}	0.317 ± 0.022^{a}	0.087 ± 0.005^{a}
Maturity stage	-Cd	-Mo	0.48 ± 0.04^{c}	0.009 ± 0.001^{c}	0.079 ± 0.004^{b}	0.042 ± 0.003^{b}
		+Mo	0.35 ± 0.04^{c}	0.005 ± 0.000^{c}	0.296 ± 0.035^{a}	0.081 ± 0.005^{a}
	+Cd	-Mo	19.82 ± 2.43^{a}	0.328 ± 0.029^{a}	0.101 ± 0.006^{b}	0.048 ± 0.004^{b}
		+Mo	14.89 ± 1.64^{b}	0.230 ± 0.022^{b}	0.335 ± 0.033^{a}	0.088 ± 0.008^{a}

-Mo and +Mo denote 0 and 0.15 mg of molybdenum supplied as ammonium molybdate [$(NH_4)_6Mo_7O_{24}\cdot 4H_2O$]), whilst -Cd and +Cd denote 0 and 100 mg of cadmium supplied as cadmium chloride ($CdCl_2\cdot 2.5H_2O$) per kg soil, respectively. The numeric values reflect means from four independent replicates (± S.E.) with different treatments. Data presented in individual columns indicated with dissimilar letters differ significantly by LSD test at $p < 0.05$.
